# Nucleoid compaction influences carboxysome localization and dynamics in *Synechococcus elongatus* PCC 7942

**DOI:** 10.1128/mbio.01919-25

**Published:** 2025-08-21

**Authors:** Claire E. Dudley, Christopher A. Azaldegui, Daniel J. Foust, Olivia LaCommare, Julie S. Biteen, Anthony G. Vecchiarelli

**Affiliations:** 1 Department of Molecular, Cellular, and Developmental Biology, University of Michigan 1259, Ann Arbor, Michigan, USA; 2 Doctoral Program in Chemical Biology, University of Michigan 1259, Ann Arbor, Michigan, USA; 3 Department of Chemistry, University of Michigan 1259, Ann Arbor, Michigan, USA; University of California Berkeley, Berkeley, California, USA

**Keywords:** ParA/MinD ATPases, subcellular organization, bacterial microcompartments, chromosome, cell stress

## Abstract

The bacterial nucleoid is not just a genetic repository—it serves as a dynamic scaffold for spatially organizing key cellular components. ParA-family ATPases exploit this nucleoid matrix to position a wide range of cargos, yet how nucleoid compaction influences these positioning reactions remains poorly understood. We previously characterized the maintenance of carboxysome distribution (Mcd) system in the cyanobacterium *Synechococcus elongatus* PCC 7942, where the ParA-like ATPase McdA binds the nucleoid and interacts with its partner protein, McdB, to generate dynamic gradients that distribute carboxysomes for optimal carbon fixation. Here, we investigate how nucleoid compaction impacts carboxysome positioning, particularly during metabolic dormancy when McdAB activity is downregulated. We demonstrate that a compacted nucleoid maintains carboxysome organization in the absence of active McdAB-driven positioning. This finding reveals that the nucleoid is not merely a passive matrix for positioning but a dynamic player in spatial organization. Given the widespread role of ParA-family ATPases in the positioning of diverse cellular cargos, our study suggests that the nucleoid compaction state is a fundamental, yet underappreciated, determinant of mesoscale organization across bacteria.

**IMPORTANCE:**

Bacteria can organize their internal components in specific patterns to ensure proper function and faithful inheritance after cell division. In the cyanobacterium *Synechococcus elongatus*, protein-based compartments called carboxysomes fix carbon dioxide and are distributed in the cell by a two-protein positioning system. Here, we discovered that when cells stop growing or face stress, these positioning proteins stop working, yet carboxysomes remain distributed in the cell. Our study shows that the bacterial chromosome, which holds genetic information, can also act as a flexible scaffold that holds carboxysomes in place when compacted. This insight reveals that the bacterial chromosome plays a key physical role in organizing the cell. Similar positioning systems are found across many types of bacteria; therefore, our findings suggest that nucleoid compaction may be a universal and underappreciated factor in maintaining spatial order in cells that are not actively growing.

## INTRODUCTION

Bacteria have evolved diverse strategies to spatially organize intracellular components. The ParA family of positioning ATPases is widespread across the bacterial kingdom, regulating a variety of cellular cargos, including chromosomes, plasmids, protein-based complexes, and organelles (1–6). Regardless of the cargo type, the bacterial nucleoid serves as the positioning matrix. Upon ATP binding, ParA ATPases dimerize, enabling them to bind nucleoid DNA (7–11). This dimerization also creates a binding site for an ATPase-activating partner protein that associates with the cargo, locally stimulating ParA ATPase activity and promoting its release from the nucleoid (6, 12, 13). This dynamic interplay generates ParA gradients that distribute cargos along the nucleoid via a Brownian ratchet mechanism (14–16). Despite current models treating the nucleoid as a passive matrix for cargo positioning (2), the nucleoid is highly dynamic, continuously compacting and decompacting in response to environmental cues (17–21). It remains unclear how these fluctuations in compaction state influence ParA-mediated cargo organization in bacterial cells.

Bacterial microcompartments (BMCs) are protein-based organelles that encapsulate key metabolic processes (22, 23). A recent bioinformatics survey identified 68 BMC types across 45 bacterial phyla (23). Despite their prevalence and metabolic significance, little is known about how BMCs are spatially regulated within the cell. The carboxysome, a carbon-fixing BMC, is the best-studied example of BMC subcellular organization (14, 24–29). Carboxysomes are widespread among cyanobacteria and some chemoautotrophs, contributing significantly to global CO_2_ fixation (14, 30), making them of significant ecological and biotechnological interest.

In the model rod-shaped β-cyanobacterium *Synechococcus elongatus* PCC 7942 (*S. elongatus* hereafter), a two-protein system ensures the even distribution of carboxysomes along the cell length during exponential growth under optimal conditions (3, 14). The maintenance of carboxysome distribution protein A (McdA) is a ParA family ATPase that binds nucleoid DNA in its ATP-bound dimeric form (3, 14, 26). Its partner protein, McdB, localizes to carboxysomes and drives McdA oscillations, which facilitate the equidistant positioning of carboxysomes (14, 25). In the absence of McdAB, carboxysomes mislocalize into nucleoid-excluded clusters, leading to their rapid loss from the cell population and slower autotrophic growth (14, 25).

Here, we investigate the role of the nucleoid and its compaction state in the spatial organization of carboxysomes. Under cellular stress, when the McdAB system is downregulated, we find that the compacted nucleoid helps maintain carboxysome organization. Additionally, our findings indicate that nucleoid compaction influences carboxysome dynamics within the cell. We propose that nucleoid compaction plays a crucial role in organizing mesoscale cellular cargos in bacteria. Specifically, we show that a compacted nucleoid can anchor distributed carboxysomes in place, even in the absence of the McdAB system. These findings have broad implications in establishing the nucleoid as an active participant—rather than a passive scaffold—in intracellular organization. They suggest that global changes in nucleoid architecture can directly influence the spatial dynamics of all cellular cargos organized by ParA family ATPases, fundamentally reshaping our understanding of bacterial cell organization and its adaptability under stress.

## RESULTS

### Carboxysomes remain distributed in stationary phase cells

We have shown that carboxysomes in *S. elongatus* are distributed along the cell length by the McdAB system in the exponential phase while under optimal growth conditions (14, 25). Cameron et al. showed that carboxysomes in stationary phase cells (~ 30 days on solid BG11 media) remain distributed (31). Intriguingly, the presence of chlorophyll indicated these carboxysome-containing cells at stationary phase remain photosynthetically active and viable, as dead cells were devoid of both chlorophyll and carboxysomes. The results suggest that carboxysome maintenance is critical to cellular fitness during extended periods of nutrient limitation. However, it remains to be determined if and how the McdAB system maintains carboxysome organization during nutrient stress.

We first set out to determine how carboxysome organization is influenced when cells transition from exponential growth to stationary phase in batch culture. We used a growth curve to determine the exponential and stationary phases of *S. elongatus* growth under our standard conditions (Fig. S1A). To image carboxysomes, the fluorescent protein monomeric Turquoise2 (mTQ) was fused to the C-terminus of the small subunit of Rubisco (RbcS) to make RbcS-mTQ, as described previously (14). *RbcS-mTQ* was expressed using a second copy of its native promoter (inserted at neutral site 1) in addition to wild-type *rbcS* at its native locus in wild-type (WT) cells. Growth of the fluorescently labeled strains was similar to that of wild-type (Fig. S1A).

**Fig 1:**
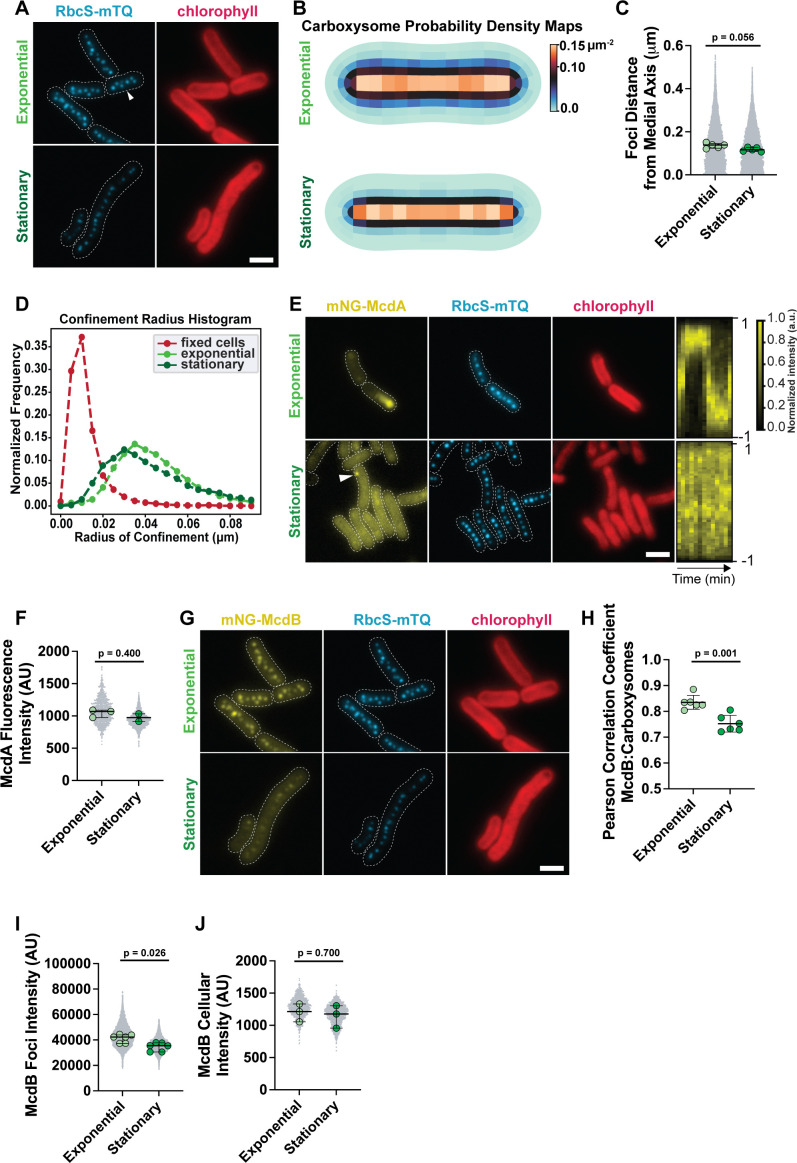
Carboxysomes are positioned in living stationary phase cells. (**A**) Representative microscopy images of carboxysomes in exponential and stationary phase cells. Carboxysomes are in cyan, and the cell boundary from the phase contrast channel is a dashed white line. White arrow highlights hexagonal packing of carboxysomes in exponential phase cells. The chlorophyll channel is in red. (**B**) Carboxysome probability density maps from cells in each growth phase. Exponential, *n* = 1,662 cells; stationary, *n* = 1,401 cells. (**C**) Carboxysome focus distance from the medial axis. Distances of carboxysome foci from the long axis of the cell, computed using the particle mapping code (see Materials and Methods). Significance from the Mann-Whitney test, *n* = 5 biological replicates for each condition, >200 cells each, bars show median and interquartile range. (**D**) Confinement radius histogram illustrating carboxysome focus confinement radii in exponential (13,937 carboxysomes), stationary (5,650 carboxysomes), and fixed cells (6,248 carboxysomes) based on TrackMate analysis. (**E**) Microscopy images of mNG-McdA and carboxysome foci in exponential and stationary phase cells. A dotted white line shows cells’ boundaries from phase contrast. Chlorophyll channel is in red. Kymographs of mNG-McdA dynamics from a representative cell in exponential and stationary phases are also provided. Short-axis is time, 0 to 60 minutes; the long-axis is cell length. (**F**) mNG-McdA whole-cell fluorescence intensity normalized by cell length. Significance from Mann-Whitney test, (exponential) *n* = 3 biological replicates and (stationary) *n* = 2 biological replicates, >200 cells each, bars show median and interquartile range. (**G**) Microscopy images of mNG-McdB in exponential and stationary phase cells. The chlorophyll (red) and carboxysome (cyan) channels are duplicate images from **A**. (**H**) Pearson’s correlation coefficient from McdB and carboxysome fluorescence channels. Significance from Welch’s *t*-test, *n* = 6 biological replicates for each condition, bars show mean and standard deviation. (**I**)Intensity of mNG-McdB foci. Significance from Mann-Whitney test, *n* = 6 biological replicates for each condition, >200 cells each, bars show median and interquartile range. (**J**) Whole-cell McdB fluorescence intensity normalized by cell length. Significance from Mann-Whitney test, *n* = 3 biological replicates for each condition, >200 cells each, bars show median and interquartile range. (scale bar, 2 µm).

As shown previously (14), RbcS-mTQ-labeled carboxysomes are distributed down the long axis of *S. elongatus* cells during exponential growth (Fig. 1A). In stationary phase, viable cells with a chlorophyll signal also had carboxysomes distributed along the cell length. However, carboxysomes were more tightly packed along the medial axis of the cell (Fig. 1B and C), with a loss in the hexagonal spacing of carboxysomes found in exponential cells (Fig. 1A, arrow) (14). The tight linear arrangement suggested that carboxysome diffusion was also restricted in stationary phase cells. Consistently, an analysis on the confinement radius of carboxysome diffusion found that carboxysome localization and movement are both restricted in stationary phase cells (Fig. 1D; Video S1 and S2). Carboxysome foci in stationary phase cells showed no significant differences in RbcS-mTQ intensity (Fig. S1B), spacing (Fig. S1C), or copy number (Fig. S1D) relative to exponential phase. Together, the data suggest that the carboxysome distribution is maintained in stationary phase cells.

### Localization of the McdAB system is perturbed during stationary phase

We have previously shown that the McdAB system is responsible for distributing carboxysomes in *S. elongatus* (14, 25). Without the McdAB system, mispositioned carboxysomes form nucleoid-excluded clusters (14, 26). Given our data showing that carboxysomes remain distributed in the stationary phase, we set out to determine if the McdAB system was responsible.

To simultaneously image McdA or McdB in our carboxysome reporter strain, McdA or McdB was N-terminally fused to the fluorescent protein monomeric NeonGreen (mNG) (32). We have previously shown that mNG-McdA and mNG-McdB are functional for carboxysome positioning when expressed as the only copy at their native locus (14). Growth of these fluorescently labeled strains was similar to that of WT (Fig. S1A). We also performed phase-contrast imaging to monitor changes in the cell morphology.

As shown previously, McdA oscillates from pole to pole on the nucleoid in *S. elongatus* cells during exponential growth (Fig. 1E; Fig. S1E; Video S3). Intriguingly, robust McdA oscillation was lost in stationary phase cells (Fig. 1E; Fig. S1F and Video S4). Instead, most stationary phase cells with a chlorophyll signal displayed diffuse McdA in the cytoplasm, despite carboxysomes remaining distributed across the cell length. A few cells also contained bright non-oscillatory McdA foci that did not colocalize with carboxysomes (Fig. 1E, arrow). We have shown previously that the mNG-McdA signal intensity is proportional to McdA levels in the cell (14, 26). Quantification of whole-cell McdA fluorescence intensity, normalized by cell length, found that the median McdA level in stationary phase cells does not decrease significantly compared to exponential-phase cells (Fig. 1F). The data suggest that McdA in stationary phase cells is no longer competent for dynamic nucleoid binding and oscillation, yet carboxysomes remain distributed.

The intensity of mNG-McdB associated with carboxysomes decreased significantly in stationary phase cells (Fig. 1G through I). Yet, quantification of whole-cell mNG-McdB fluorescence intensity, normalized by cell length, found that the median McdB level was similar to that of exponential phase cells (Fig. 1J). Together, our data show that while the cellular levels for both McdA and McdB are maintained in the stationary phase, McdA no longer associates with the nucleoid and McdB colocalizes less with carboxysomes. The evidence suggests that the McdAB system is not the major driver of carboxysome distribution in stationary phase cells, which led us to consider other factors.

### Phosphate depletion compacts the nucleoid and downregulates McdAB, yet carboxysomes remain distributed

**Fig 2:**
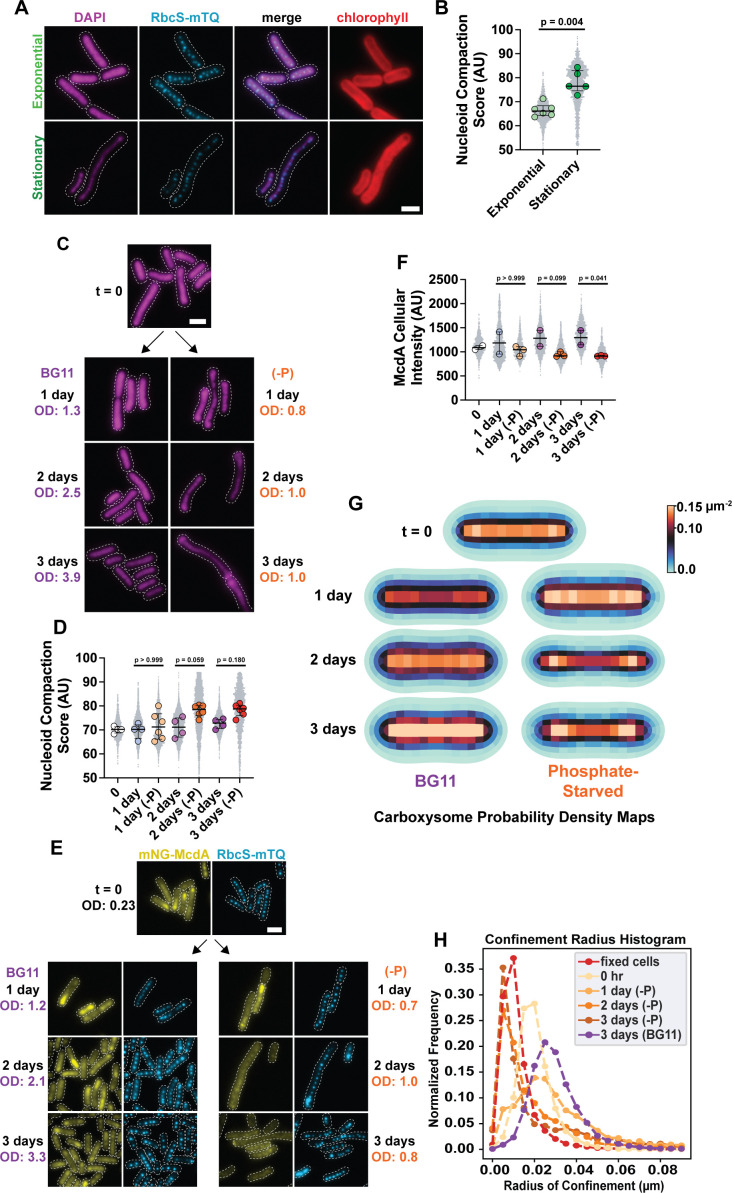
Phosphate deprivation induces nucleoid compaction and downregulation of McdAB. (**A**) Microscopy images of DAPI-stained nucleoids (magenta) in exponential and stationary phase cells. A dotted white line shows cell boundaries from phase contrast. Carboxysomes are in cyan, and the merge shows carboxysome and DAPI channels. Red is the chlorophyll channel. The chlorophyll (red) and carboxysome (cyan) channels are duplicate images from Fig. 1A. (**B**) Nucleoid compaction score in arbitrary units (see Materials and Methods). Significance from Mann-Whitney test, (exponential) *n* = 6 biological replicates and (stationary) *n* = 5 biological replicates, >200 cells per replicate, bars show median and interquartile range. (**C**) Microscopy images of DAPI-stained nucleoids in cells grown in BG11 or phosphate-depleted (-P) media. Dotted white line shows cell boundaries from phase contrast. (**D**) Nucleoid compaction score in arbitrary units in non-starved and phosphate-limited (-P) cells. Significance from Kruskal-Wallis and Dunn’s multiple comparisons. Biological replicates, (BG11) *n* = 4 and (-P) *n* = 6, >200 cells per replicate, bars show median and interquartile range. (**E**) Microscopy images of mNG-McdA and carboxysome foci in cells grown in BG11 and phosphate-limited BG11 (-P) media over a 3-day time course. Dotted white line shows cell boundaries from phase contrast. (**F**) mNG-McdA whole-cell fluorescence intensity normalized by cell length. Significance from Kruskal-Wallis and Dunn’s multiple comparisons. Biological replicates, (BG11) *n* = 2 and (-P) *n* = 3, >300 cells per replicate, bars show median and interquartile range. (**G**) Carboxysome probability density map from cells grown in BG11 and phosphate deprivation conditions. *t* = 0, *n* = 1,037 cells; 1 day: *n* = 629 cells (BG11), *n* = 1,216 cells (-P); 2 days: *n* = 1,079 cells (BG11), *n* = 1,490 cells (-P); 3 days: *n* = 1,857 cells (BG11), *n* = 1,317 cells (-P). (**H**) Confinement radius histogram illustrating carboxysome diffusion radii in cells grown in the conditions indicated: fixed: 6,248 carboxysomes, 0 hr: 8,394 carboxysomes, 1 day (-P): 1,737 carboxysomes, 2 days (-P): 2,179 carboxysomes, 3 days (-P): 2,770 carboxysomes, 3 days (BG11): 4,586 carboxysomes. (scale bar, 2 µm).

The nucleoid serves as a critical matrix for positioning diverse cellular cargos by ParA family ATPases, including chromosomes, plasmids, and an array of protein-based organelles, many of which are fundamental to cell survival and pathogenesis (2, 33, 34). Yet it remains to be determined how the compaction state of the nucleoid influences the subcellular organization of these mesoscale cargos. Consistent with other bacterial species (18, 19, 35), the nucleoid of *S. elongatus* significantly compacts in stationary phase (Fig. 2A and B). Nucleoid compaction correlated with carboxysomes aligning down the short axis of stationary phase cells (Fig. 2A). Together, the data suggest that the compacted nucleoid holds distributed carboxysomes in place in stationary phase cells when the McdAB system is downregulated.

Based on this finding, we set out to determine the specific conditions in stationary phase driving nucleoid compaction. It has been shown previously that phosphate depletion induces nucleoid compaction in *S. elongatus* (17, 36). Indeed, cells grown in phosphate-free BG11 media exhibited significant nucleoid compaction, compared to cells grown in phosphate-sufficient media (Fig. 2C and D). The degree of nucleoid compaction after 2 days of phosphate starvation was comparable to that found in stationary-phase cells (see Fig. 2B). Phosphate starvation also halted cell growth (Fig. S2A). The data suggest that phosphate depletion serves as a key driver of nucleoid compaction and the resulting alignment of carboxysomes in stationary phase cells.

Observing these significant changes in the topology of the positioning matrix under phosphate depletion led us to consider how McdA oscillation and carboxysome distribution are affected. We found that McdA oscillations dampened after 1 day of phosphate depletion and were largely undetectable after 3 days (Fig. 2E; Fig. S2B). McdA cellular levels were also lower in phosphate-depleted cells compared to cells grown in BG11 (Fig. 2F). Despite the decrease in McdA levels and loss of McdA oscillations, carboxysome focus number, intensity, and spacing did not undergo drastic changes (Fig. S2C through E). Also, McdB colocalization and intensity on carboxysomes did not significantly change over the 3-day phosphate starvation period (Fig. S2F and G). However, as in stationary phase cells, carboxysomes were better aligned along the medial axis in phosphate-starved cells (Fig. 2G).

Interestingly, in phosphate-deprived cells showing areas of both nucleoid expansion and compaction, carboxysomes were linearly distributed along the compressed stretches of nucleoid and clustered in areas of expansion (Fig. S2H). Consistent with this observation, the degree of nucleoid compaction in phosphate-deprived cells also correlated with restricted carboxysome diffusion in the cell (Fig. 2H)—after 2 days of phosphate deprivation, carboxysomes were essentially static in the cell. Together, we conclude that phosphate depletion results in the downregulation of the McdAB system and nucleoid compaction, which immobilizes carboxysomes and maintains their linear distribution in the absence of active positioning.

### Under prolonged darkness, McdA is sequestered, and the nucleoid does not compact, resulting in carboxysome clustering

**Fig 3:**
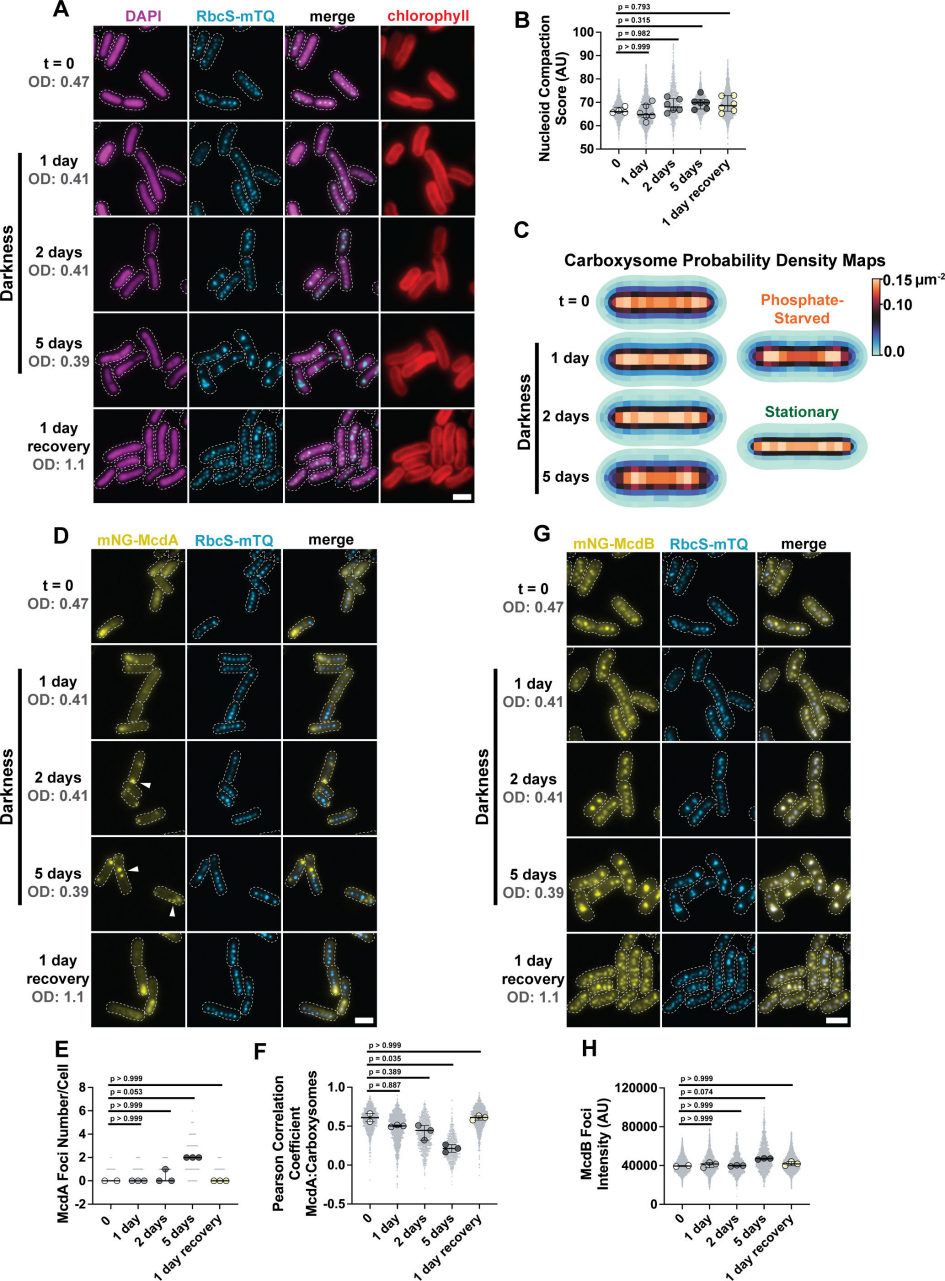
McdA is sequestered, and carboxysomes cluster in prolonged darkness. (**A**) Microscopy images of DAPI-stained nucleoids (magenta) and carboxysomes (cyan) in cells grown in light deprivation conditions over 5 days and 1 day continuous light recovery. A dotted white line shows cell boundaries from phase contrast. Chlorophyll channel is red. (**B**) Nucleoid compaction score in arbitrary units. Significance from Kruskal-Wallis and Dunn’s multiple comparisons, *t* = 0, *n* = 4 biological replicates; days 1–5 and 1 day recovery, *n* = 6 biological replicates per time point, >200 cells per replicate, bars show median and interquartile range. (**C**) Carboxysome probability density map from cells grown in prolonged darkness. *t *= 0, *n* = 840 cells; 1 day, *n* = 845 cells; 2 days, *n* = 376 cells; 5 days, *n* = 736 cells; 1 day recovery, *n* = 1,961 cells; 3 days phosphate starvation, *n* = 1,317 cells; stationary, *n* = 1,401 cells (**D**) Microscopy images of mNG-McdA and carboxysomes (cyan) in cells grown in light deprivation conditions over 5 days and 1 day continuous light recovery. Dotted white line shows cell boundaries from phase contrast. White arrows highlight non-oscillatory foci. (**E**) mNG-McdA focus number per cell. Significance from Kruskal-Wallis and Dunn’s multiple comparisons, *t* = 0, *n* = 2 biological replicates; days 1–5 and 1 day recovery, *n* = 3 biological replicates per time point, >200 cells per replicate, bars show median and interquartile range. (**F**) Pearson correlation coefficient from McdA and carboxysome fluorescence channels. Significance from Kruskal-Wallis and Dunn’s multiple comparisons, *t* = 0, *n* = 2 biological replicates; days 1–5 and 1 day recovery, *n* = 3 biological replicates per time point, >200 cells per replicate, bars show median and interquartile range. (**G**) Microscopy images of mNG-McdB and carboxysomes (cyan) in cells grown in light deprivation conditions over 5 days and 1 day continuous light recovery. Dotted white line shows cell boundaries from phase contrast. The carboxysome (cyan) channel is a duplicate image from panel **A**. (**H**) Intensity of McdB foci. Significance from Kruskal-Wallis and Dunn’s multiple comparisons, *t* = 0, *n* = 2 biological replicates; days 1–5 and 1 day recovery, *n* = 3 biological replicates per time point, >200 cells per replicate, bars show median and interquartile range (scale bar, 2 µm).

The circadian clock of *S. elongatus* has been shown to control nucleoid compaction—compacting at dusk and expanding through the night, effectively regulating gene expression through these physical changes in chromosome structure (37). However, the effects of prolonged light limitation due to high cell densities at stationary phase in batch culture or in the environment have not been investigated. Therefore, to determine if light limitation also influences nucleoid compaction, we incubated cells in continuous darkness for 5 days. During this time, growth halted (Fig. S3A). Even after 5 days in darkness, the nucleoid did not dramatically compact compared to cells grown in constant light (Fig. 3A and B). Reintroduction into continuous light for 1 day recovered cell growth (Fig. S3A). The data show that prolonged darkness halts *S. elongatus* growth, but this effect is not coupled to the drastic changes in nucleoid compaction we observed in stationary phase or phosphate-starved cells.

Prolonged darkness, therefore, provided an opportunity to observe carboxysome organization in non-growing cells without a highly compacted nucleoid. Unlike in the stationary phase or phosphate-starved cells, carboxysome foci did not align down the medial axis of cells in prolonged darkness (Fig. 3A and C). Instead, carboxysomes clustered into fewer (Fig. S3B), high-intensity foci (Fig. S3C) that were more distantly spaced from one another (Fig. S3D). Strikingly, carboxysomes redistributed within 1 day of light reintroduction (Fig. 3A; Fig. S3B through D). The data show that prolonged darkness results in carboxysomes forming mispositioned clusters similar to cells lacking a functioning McdAB system.

Since the data suggested an inactive McdAB system, we imaged McdA and McdB localization under continuous darkness. Cellular levels of McdA declined by 25% after 5 days in darkness (Fig. S3E), and intriguingly, McdA oscillations transitioned into punctate foci, which coincided with carboxysomes forming mispositioned clusters in the cell (Fig. 3D and E). The McdA foci were static (Fig. S3F) and did not colocalize with mispositioned carboxysomes (Fig. 3F). McdB, on the other hand, remained associated with the mispositioned carboxysome clusters over prolonged darkness (Fig. 3G and H). One day of light reintroduction was sufficient to recover both McdA oscillation and carboxysome redistribution across the cell population (Fig. 3; Fig. S3). Together, we conclude that under prolonged darkness, McdA is sequestered into foci and the nucleoid remains expanded, which allows carboxysomes to diffuse and cluster in the absence of active positioning by the McdAB system.

### Drug-induced nucleoid compaction overrides the McdAB system in maintaining carboxysome distribution

**Fig 4:**
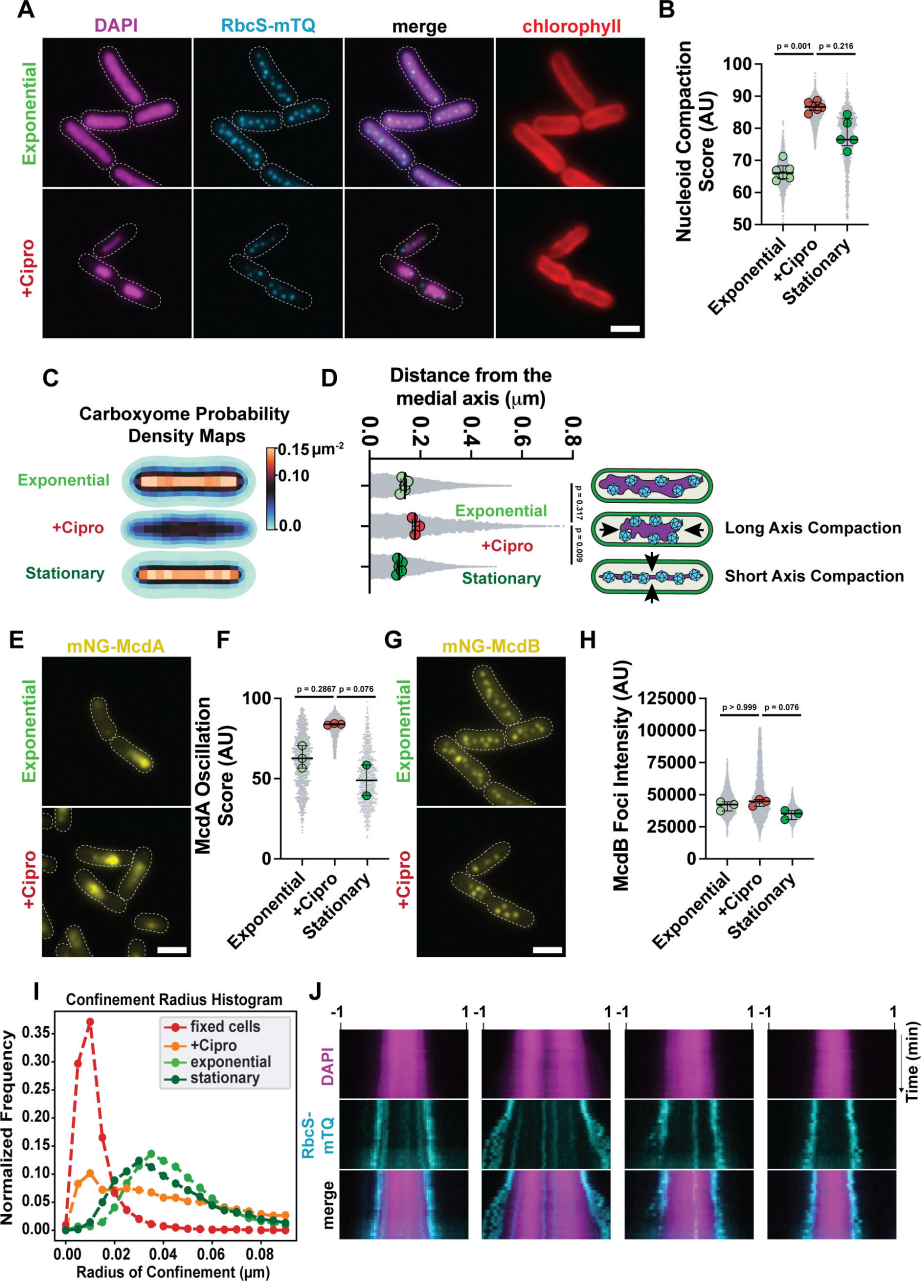
Drug-induced nucleoid compaction overrides the McdAB system in maintaining carboxysome distribution. (**A**) Microscopy images of DAPI-stained nucleoids and carboxysomes (cyan) in exponential growth with or without 50 µM ciprofloxacin for 4 hours. Red is the chlorophyll channel. The untreated exponential chlorophyll (red) and carboxysome (cyan) images are duplicates of Fig. 1A. The DAPI (magenta) and merge images are duplicates of Fig. 2A. (**B**) Nucleoid compaction score in arbitrary units. Significance from Kruskal-Wallis and Dunn’s multiple comparisons, (exponential) *n* = 6 biological replicates, (cipro) *n* = 6 biological replicates, and (stationary) *n* = 5 biological replicates, >200 cells per replicate, bars show median and interquartile range. (**C**) Carboxysome probability density maps from cells in the exponential phase, exponential cells treated with ciprofloxacin, and stationary phase. Exponential, *n* = 1,662 cells; ciprofloxacin, *n* = 969 cells; stationary, *n* = 1,401 cells. (**D**) mTQ focus distance from the medial axis. Distances of mTQ foci from the long axis of the cell, computed using the particle mapping code. Significance from Kruskal-Wallis and Dunn’s multiple comparisons, (exponential) *n* = 6 biological replicates, (cipro) *n* = 6 biological replicates, and (stationary) *n* = 5 biological replicates, >200 cells per replicate, bars show median and interquartile range. (**E**) Microscopy images of mNG-McdA in cells with and without ciprofloxacin treatment. The untreated mNG-McdA image is a duplicate of Fig. 1E. (**F**) McdA oscillation score demonstrating oscillatory (higher score) or diffuse nature (lower score) in arbitrary units. Significance from Kruskal-Wallis and Dunn’s multiple comparisons, (exponential) *n* = 3 biological replicates, (cipro) *n* = 3 biological replicates, and (stationary) *n* = 2 biological replicates, >200 cells per replicate, bars show median and interquartile range. (**G**) Microscopy images of mNG-McdB in cells with and without ciprofloxacin treatment. The untreated mNG-McdB image is a duplicate of Fig. 1G. (**H**) Intensity of McdB foci. Significance from Kruskal-Wallis and Dunn’s multiple comparisons, (exponential) *n* = 3 biological replicates, (cipro) *n* = 3 biological replicates, and (stationary) *n* = 3 biological replicates, >200 cells per replicate, bars show median and interquartile range. (**I**) Confinement radius histogram illustrating carboxysome focus confinement radii in exponential (13,937 carboxysomes), exponential treated with ciprofloxacin (6,261 carboxysomes), stationary (5,650 carboxysomes), and fixed cells (6,248 carboxysomes) based on TrackMate analysis. (**J**) Representative kymographs of DAPI-stained nucleoids and carboxysomes (cyan) in ciprofloxacin-treated cells. Long-axis is cell length, short-axis is time, 0 to 60 minutes. (scale bar, 2 µm).

Nucleoid compaction is one of several physiological changes that occur when bacterial growth conditions are altered. Therefore, the data presented thus far are correlative—showing a strong correlation between nucleoid compaction and the maintenance of carboxysome distribution in the absence of active positioning by the McdAB system. We next set out to determine if drug-induced nucleoid compaction directly influenced carboxysome distribution. We have previously shown that the gyrase inhibitor, ciprofloxacin, compacts the nucleoid in *S. elongatus* (26). We find here that when exponential cells were treated with ciprofloxacin, carboxysomes remained distributed over hypercompacted nucleoids in viable cells (Fig. 4A; Fig. S4A through C). The term “hyper-compacted” is used to emphasize that the degree of nucleoid compaction is greater than that in stationary-phase cells (Fig. 4B). Additionally, the hypercompacted nucleoids were largely compacted along the long axis of cells, whereas nucleoids were compacted along the short axis in our nutrient deprivation experiments (Fig. 4C). This hypercompaction of the nucleoid resulted in carboxysomes being positioned further away from the medial axis of the cell (Fig. 4D). Together, the data show that nucleoid compaction directly influences the distribution of carboxysomes.

We next set out to determine the localization and dynamics of the McdAB system in cells with hyper-compacted nucleoids. Interestingly, McdA displayed robust oscillation over the shortened nucleoid region of the cell (Fig. 4E and F; Fig. S4D; Video S5). McdB also remained localized to carboxysomes (Fig. 4G), albeit with a broader intensity distribution (Fig. 4H); likely due to space limitations for distributing carboxysomes on a hyper-compacted nucleoid. Despite McdA oscillation and McdB colocalization with carboxysomes being relatively unperturbed, carboxysome dynamics were severely restricted on hyper-compacted nucleoids, more so than on the compacted nucleoids of stationary phase cells (Fig. 4I). The findings show that a hypercompacted nucleoid can override a functional McdAB system to maintain carboxysome organization in the cell. Consistent with this interpretation, carboxysomes near the poles of hypercompacted nucleoids became more dynamic over time as polar regions of the nucleoid re-expanded following drug removal (Fig. 4J).

## DISCUSSION

**Fig 5:**
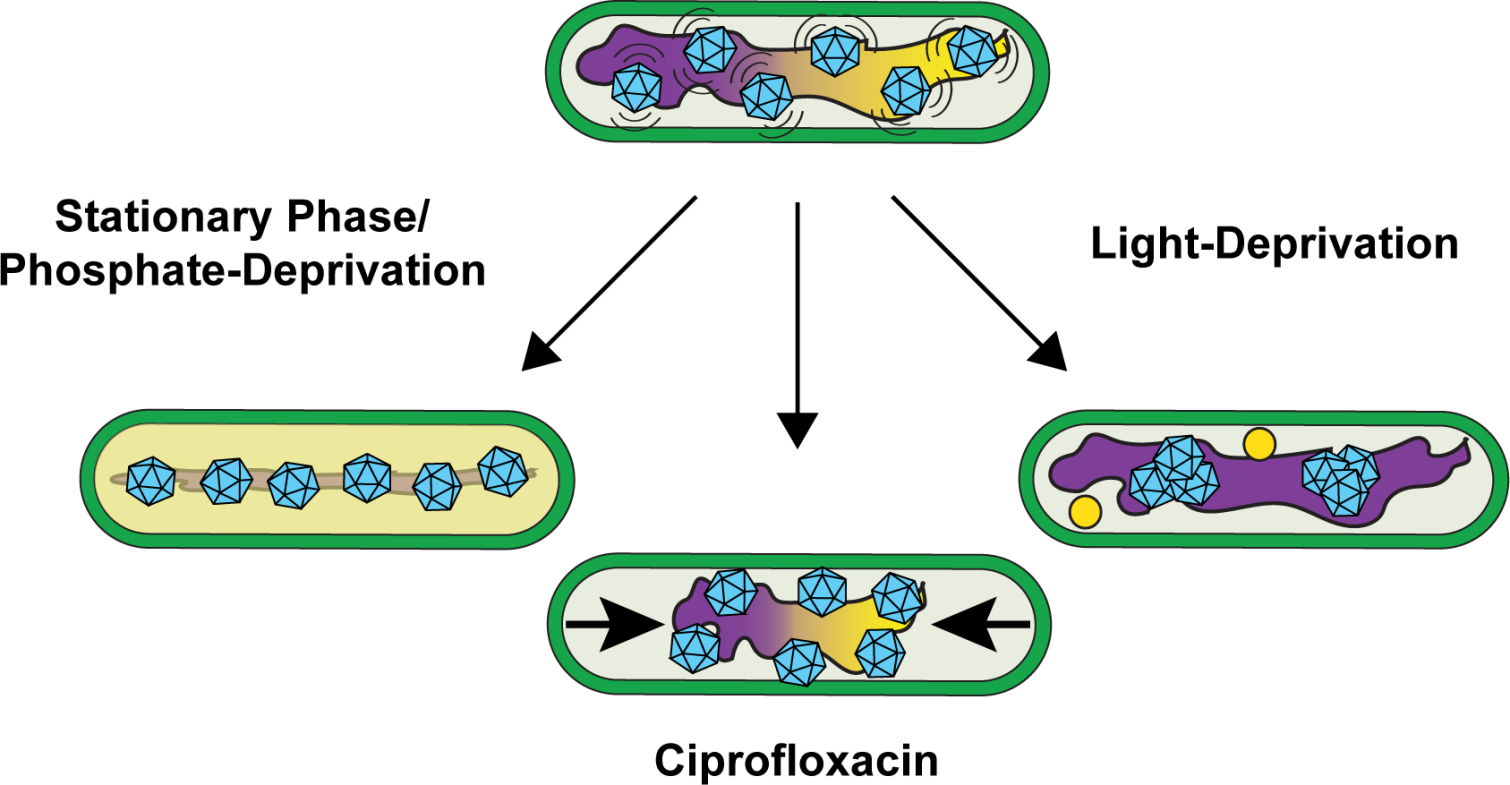
The compacted nucleoid can maintain carboxysome distribution. McdA oscillation (yellow) on the nucleoid (magenta) distributes McdB-bound carboxysomes (cyan) in exponential phase cells (top). In the stationary phase or upon phosphate depletion (left), static carboxysomes are tightly aligned along the medial axis, despite a loss of McdA oscillation. Ciprofloxacin treatment resulted in hypercompacted nucleoids that immobilized carboxysomes, which were unresponsive to McdA oscillation (*bottom*). With light deprivation (right), McdA was sequestered, resulting in carboxysomes clustering on an expanded nucleoid.

The nucleoid serves as a critical matrix for positioning diverse cellular cargos by ParA family ATPases, including chromosomes, plasmids, and an array of protein-based organelles, many of which are fundamental to cell survival and pathogenesis (2, 24, 33, 34). Yet it remains unclear how the compaction state of the nucleoid influences the subcellular organization of these mesoscale cargos. In this study, we investigated the mechanisms underlying carboxysome organization in *S. elongatus* under physiological states that influence nucleoid compaction, particularly during stationary phase and under environmental stresses such as phosphate deprivation and prolonged darkness. We found that, while carboxysomes remain distributed in stationary phase cells, their spatial arrangement is altered, becoming more tightly aligned along the medial axis with restricted diffusion. Surprisingly, this distribution was maintained despite the loss of McdA oscillation and the reduced colocalization of McdB with carboxysomes, suggesting that factors other than the McdAB system contribute to carboxysome positioning during stationary phase. We further demonstrated that nucleoid compaction, induced by phosphate starvation or drug treatment, correlates with carboxysome immobilization and alignment along the axis of nucleoid compaction, while prolonged darkness led to McdA sequestration and carboxysome clustering on an expanded nucleoid (Fig. 5). Together, our findings reveal that the nucleoid structure plays a significant role in carboxysome positioning, particularly under conditions where the McdAB system is downregulated or inactivated.

### Carboxysome distribution and suppression of McdAB in non-growing cells

Our results support and extend previous observations that carboxysomes remain distributed in stationary phase cells (31). However, in contrast to exponential phase cells, where McdAB actively positions carboxysomes (14, 25, 27), we find that McdA loses its dynamic oscillation and McdB no longer strongly colocalizes with carboxysomes in the stationary phase. Despite these differences, carboxysomes remain evenly distributed along the cell length, albeit in a more compacted linear arrangement. The data implicate the compacted nucleoid in maintaining carboxysome positioning when McdAB activity is diminished.

The decreased function of McdA and McdB proteins may stem from the metabolic shifts associated with stationary phase. McdA and McdB levels did not significantly decline in stationary phase cells; therefore, their functional impairment and mislocalization in the cell suggest regulatory control at the post-translational level. A decrease in the cellular ATP pool is one plausible mechanism for McdA inactivation. ATP depletion is a well-documented consequence of prolonged starvation and energy stress in bacteria (38–40). Since McdA requires ATP binding to dimerize and interact with the nucleoid, a reduction in ATP availability could prevent the formation of active McdA dimers, thereby abolishing oscillations on the nucleoid. Our observation that McdA oscillations gradually dampen and become undetectable during phosphate starvation, or after prolonged darkness, is consistent with a model in which ATP depletion leads to McdA inactivation.

Interestingly, McdA also formed static foci in darkness that did not colocalize with mispositioned carboxysomes. It is possible that, in the absence of sufficient ATP, McdA may misfold or aggregate into inactive assemblies. This type of ATP-dependent aggregation has been observed for other ATPases under energy-limited conditions and may serve as a protective mechanism to prevent protein degradation during prolonged darkness stress (41–43). The rapid recovery of McdA oscillation and carboxysome redistribution upon re-exposure to light suggests that once ATP levels are restored, McdA aggregates dissolve, allowing McdA dimers to resume function.

Another possible explanation for McdAB inactivation in non-growing cells is a stress-induced drop in cytoplasmic pH. Some bacterial stress responses, including those triggered by light deprivation, lead to cytoplasmic acidification, which can alter protein structure, stability, and function (44–46). Given that McdA requires ATP binding for dimerization and nucleoid association, a reduction in cytoplasmic pH could directly impact its ATPase activity, oligomeric state, and nucleoid-binding ability.

McdB colocalization with carboxysomes significantly declined in stationary phase cells, yet remained strongly colocalized during phosphate depletion or prolonged darkness. The trigger for this growth-phase-specific release of McdB release remains to be determined. McdB interacts with carboxysome shell proteins (14), but the nature of this interaction is unknown. We previously found that McdB forms pH-regulated condensates, forming at pH ≤7.5 and dissolving at alkaline pH (≥8) (47). This pH transition point is intriguing because metabolically active carboxysomes, which accumulate protons during CO_2_ fixation, are thought to have a slightly acidic interior, relative to the alkaline cytoplasm of *S. elongatus* (44). It is attractive to speculate that McdB preferentially condenses on metabolically active carboxysomes (low pH) and less so on inactive ones (higher pH). Such pH-regulated condensation could allow McdB to coat or scaffold the carboxysome shell in a state-dependent manner.

### Nucleoid compaction as a driver of carboxysome organization

Our findings indicate that the nucleoid structure plays a key role in maintaining carboxysome organization, particularly when McdAB-based positioning activity is downregulated. We observed that in stationary phase, the nucleoid significantly compacts along the medial axis of the cell, and this compaction correlated with the linear alignment and restricted diffusion of carboxysomes (Fig. 5). Similarly, phosphate starvation, which induced nucleoid compaction, resulted in carboxysomes adopting a comparable medial alignment. Together, the evidence suggests that as the nucleoid compacts, it traps carboxysomes and restricts movement, effectively maintaining their spatial distribution without the need for McdAB activity.

Further supporting this idea, we found that cells undergoing partial nucleoid compaction due to phosphate starvation exhibited distinct spatial patterns—carboxysomes aligned within compacted regions, while clustering was observed in expanded areas. Additionally, ciprofloxacin-induced nucleoid hyper-compaction resulted in carboxysomes being positioned further from the medial axis, highlighting the influence of nucleoid topology on carboxysome organization. These observations demonstrate that nucleoid architecture is a major determinant of carboxysome positioning in *S. elongatus*, particularly under conditions where the McdAB system becomes inactive.

It is important to note that *S. elongatus* is a polyploid organism, with individual cells carrying between two and ten copies of the chromosome within the same population. In other polyploid cyanobacteria, such as *Synechocystis* sp. PCC 6803, which can carry over 100 genome copies, phosphate starvation has been shown to reduce chromosome copy number, suggesting that DNA may serve as a phosphorus reserve under nutrient-limited conditions (48). Several observations in our study, however, suggest that phosphate deprivation does not substantially reduce total DNA content in a way that alters our conclusions. First, DAPI staining revealed a dramatic increase in nucleoid compaction under phosphate starvation, yet the DAPI signal remained strong and broadly distributed, indicating that DNA content persists. Importantly, the compacted nucleoid was sufficient to immobilize carboxysomes, similar to what we observed in stationary phase cells. In contrast, carboxysomes remained mobile and clustered in cells with an uncompacted nucleoid under prolonged darkness. Together, these findings support the interpretation that nucleoid structure—rather than absolute DNA content—is the dominant factor controlling carboxysome positioning under stress. While potential reductions in chromosome copy number under phosphate limitation cannot be ruled out, they are unlikely to impact our central conclusion: that nucleoid compaction provides a passive mechanism for maintaining carboxysome organization when McdAB activity is diminished. How chromosome copy number influences carboxysome positioning by the McdAB system in polyploid bacteria is an important topic of future study.

### The effects of prolonged darkness on carboxysome positioning

In contrast to nutrient limitation, prolonged darkness led to a distinct response in carboxysome organization—the nucleoid remained expanded and carboxysome distribution was not maintained. Instead, carboxysomes clustered into fewer, high-intensity foci—a phenotype found when the McdAB system is nonfunctional (14, 25, 26, 28, 29). These findings suggest that prolonged darkness induces a metabolic state that disrupts McdAB function, leading to carboxysome mispositioning and clustering. This mispositioning was accompanied by McdA sequestration into static foci. Interestingly, upon re-exposure to light, both McdA oscillations and carboxysome distribution were rapidly restored, demonstrating the dynamic nature of these changes. The findings suggest that while a compacted nucleoid can serve as a passive organizing force, carboxysome distribution in the cell requires the McdAB system when the nucleoid is expanded.

### Nucleoid compaction can override the McdAB system

Our results with ciprofloxacin treatment provide strong evidence that nucleoid compaction can override active carboxysome positioning by McdAB. Despite McdA continuing to oscillate and McdB remaining associated with carboxysomes, carboxysome diffusion was severely restricted on hypercompacted nucleoids. This demonstrates that the nucleoid structure alone can dictate carboxysome positioning, even when McdAB remains functional.

Furthermore, we observed that as the nucleoid re-expanded following drug removal, carboxysome dynamics recovered, particularly at the expanding nucleoid poles. This suggests that nucleoid expansion allows carboxysomes to regain mobility and dynamic positioning by the McdAB system. These findings emphasize the hierarchical relationship between nucleoid structure and McdAB-mediated positioning, where nucleoid compaction can act as a dominant factor in determining carboxysome organization under specific conditions.

### Conclusions and future directions

Together, our findings reveal a complex interplay between active and passive mechanisms governing carboxysome organization in *S. elongatus*. While the McdAB system is essential for initially positioning carboxysomes on expanded nucleoids, our data suggest that nucleoid compaction can passively maintain carboxysome distribution during periods when McdAB is inactive. Importantly, the McdAB system is required to first localize carboxysomes within the nucleoid region. In its absence, carboxysomes form nucleoid-excluded aggregates and are unaffected by changes in nucleoid compaction. Only once carboxysomes are distributed over the nucleoid by McdAB can compaction serve to retain them in place.

These findings raise several important questions. How is the McdAB system inactivated under different stress conditions? What molecular mechanisms link nucleoid compaction to carboxysome positioning? Future studies investigating the regulatory pathways controlling McdAB function and the biophysical properties of nucleoid-compaction-driven carboxysome organization will provide valuable insights into bacterial organelle positioning strategies.

ParA-mediated partitioning of F plasmids has been shown to highly correlate with dense regions of the bacterial nucleoid (49), which is consistent with the nucleoid compaction state providing an additional layer of spatial regulation. Given the shared mesoscale sizing of the diverse cellular cargos positioned by ParA family ATPases, it is highly likely that our findings extend beyond carboxysomes and apply more broadly to other macromolecular complexes and organelles that rely on the bacterial nucleoid as a matrix for spatial organization in the cell.

## MATERIALS AND METHODS

### Constructs used

Constructs used are described in Table 1.

**TABLE 1:** Constructs used

Strain	Identifier	Description	Reference
mNG-McdA +RbcS-mTQ	C5/AH3	mNG-McdA at the native locus with the native promoter, RbcS-mTQ at neutral site I with the native promoter	(14)
mNG-McdB +RbcS-mTQ	C28/AH17	mNG-McdB at the native locus with the native promoter, RbcS-mTQ at neutral site I with the native promoter	(14)

For native McdA fluorescent fusions, the fluorescent protein mNeonGreen (mNG) was attached to the 5′ region of the native *mcdA* coding sequence, separated by a GSGSGS linker. Since the upstream coding sequence next to *mcdA* is essential and presumably expressed from the same region of DNA as *mcdA*, the kanamycin resistance cassette was inserted upstream of the *mcdA* promoter to prevent operon disruption, and a duplicate *mcdA* promoter was inserted upstream of kanamycin to drive the expression of the essential coding sequence. For the native mNG-McdB construct, the *mcdB* sequence was codon-optimized to prevent recombination at this site, and mNG was inserted at the 5′ end. The kanamycin resistance cassette was inserted downstream. To visualize carboxysomes, a second copy of the *rbcS* promoter and gene, attached at the 3′ end with the fluorescent protein mTurquoise2 (mTQ) and separated with a GSGSGS linker, was inserted into neutral site 1.

### Growth conditions

All strains were grown in BG11 media pH 8.3 (Sigma). Cultures were grown at a 50 mL volume in 125 mL beveled flasks (Corning) or at 100 mL volume in 250 mL beveled flasks. Strains were grown in a Minitron incubator (Infors-HT) at 30℃, 2% CO_2_, shaking at 130 RPM, with 60 µmol m^−2^ s^−1^ LED light. Cultures were maintained by back-diluting regularly into fresh BG11 media.

### Growth phase

Stationary-phase cultures were created by allowing an exponential culture to continue to grow for several weeks without back dilution. OD_750_ > 10 was considered stationary phase based on our growth curve.

### Nutrient deprivation

For phosphate deprivation conditions, K_2_HPO_4_ was removed from the BG11 media. Nutrient deprivation cultures were started from 100 mL exponentially growing cultures that were centrifuged at 4,000 RPM for 10 min. After decanting the supernatant, pellets were resuspended in residual media, ~1 m BG11. A volume of 100–200 µL of the resuspended pellet was then added to fresh BG11 media or BG11 media lacking phosphate to reach an initial OD_750_ of 0.1–0.2. At each time point, 2 mL of the culture was removed for imaging. Each strain was grown in triplicate, and the error bars in the data represent the standard deviation across three flasks. Images are representative across all biological replicates.

### Light deprivation

Light deprivation cultures were started from 50 mL exponentially growing cultures grown in continuous light. The cultures were split into two flasks and diluted with 25 mL fresh BG11 to reach an initial OD_750_ of 0.4–0.5. Cultures grown in darkness had no light source, and the incubator window was blocked with cardboard. At each time point, 2 mL of the culture was removed for imaging. Cultures were recovered by transferring them back to continuous light conditions for 1 day. Experiments were performed twice and across three biological replicates. Images are representative of all biological replicates.

### Ciprofloxacin treatment

Twenty-five milliliters of cells in a 125 mL beveled flask was treated with 50 µM ciprofloxacin for 4 hours to induce nucleoid compaction. To visualize the compacted nucleoid region, 2 mL of ciprofloxacin-treated *S. elongatus* cells was harvested by centrifugation at 16,000 × *g* for 1 min. The pelleted cells were then washed and resuspended in 100 µL of phosphate-buffered saline (pH 7.2).

### Growth curve measurements

Growth curve cultures were started from 100 mL exponentially growing cultures that were centrifuged at 4,000 RPM for 10 min. After decanting the supernatant, pellets were resuspended in residual media, ~1 mL BG11. A volume of 100–200 µL of the resuspended pellet was then added to fresh BG11 media to reach an initial OD_750_ of 0.1–0.2. At each time point, the OD_750_ was measured using a DS-11 spectrophotometer (Denovix). Each strain was grown in triplicate with the error bars representing the standard deviation across the three flasks.

### DAPI staining

Two milliliters of the culture was centrifuged at 16,000 × *g* for 1 min. Cells were then washed with 1× PBS (pH 7.2) and then resuspended in 100 µL of PBS. DAPI (80 µL from a 20 µg/mL stock concentration) was added to the cell suspension followed by 20 min incubation in the dark at 30°C, 2% CO_2_, and shaking at 130 RPM. DAPI-stained cells were washed twice with 1 mL H2O and then resuspended in 100 µL H_2_O prior to visualization using the DAPI channel.

### Live fluorescence microscopy

After DAPI staining, 1 µL of cells was plated on a disk made of 1.5% UltraPure agarose (Invitrogen) in BG11, which was then placed on a 35 mm cover glass-bottom dish (MatTek). Imaging was performed through the NIS Elements software on a Nikon Ti2-E inverted microscope with a 100× objective lens, SOLA LED light source, and a Hamamatsu camera. mNG-McdA and mNG-McdB were imaged using a YFP filter, RbcS-mTQ carboxysomes with a CFP filter, and DAPI stained DNA was visualized with a DAPI filter.

### Data analysis

Outliers were removed from all graphs using the ROUT method in GraphPad Prism. Technical replicates (in gray on graphs) were pooled, and the means or medians of the biological replicates (in colored circles on graphs) were used to perform statistical analysis.

### Image analysis

#### TrackMate

Carboxysomes were detected and tracked using the FIJI plugin TrackMate (50, 51). The settings for the LoG detector were an estimated blob diameter of 3.0 pixels and a threshold of 80.0. The Simple LAP tracker was used with a Linking Max Distance of 7.5 pixels, a Gap-Closing Max Distance of 4.5 pixels, and a Gap-Closing Max Frame Gap of 2. The Spots in Tracks Statistics file was used in downstream analysis.

#### Confinement radius

The first ten localizations of each trajectory were used to calculate the confinement radius. The confinement radius was determined by finding the average *xy* position of the cropped track by averaging the track coordinates. The average of the difference between this position and the track coordinates gives the confinement radius.

#### Cell registration

Phase-contrast images were registered to the first frame in the stack using cross-correlation. If the stack contained multi-channel data, the shifts used for the phase contrast images were applied to all other time-corresponding frames in other channels. The registered stacks were then saved as separate TIFF stacks.

#### Cell segmentation

Drift-corrected phase-contrast images were used for cell segmentation via the Omnipose (52) package in Python. A custom Python script was implemented for batch segmentation: phasemasks_omni.py. Prior to segmentation, a Gaussian blur (standard deviation of Gaussian = 66 nm) was applied to the images. The “bact_phase” pre-trained model was used for segmentation. Cells touching the image borders were ignored, and erroneous segmentations were manually corrected using the Omnipose GUI or excluded from further analysis if cell boundaries were not clear from the phase-contrast image. Some cell masks had gaps within the inner region of the cell representing the chlorophyll localization pattern. We corrected these errors using the binary_fill_holes function from the scipy.ndimage package (53). Resulting masks were used for subsequent single-cell analyses. For cell morphology analysis, the cell mask was used to determine the cell length, width, and area.

#### Fluorescent focus detection

Carboxysome, McdA, and McdB foci were detected using a custom Python script. First, the cell masks were used to individually analyze every cell in the field of view. The fluorescence signal in each cell was then sharpened using the scikit-image (54) function “filters.unsharp_mask”; this step was followed by a Gaussian blur. Using this preprocessed image, fluorescent foci were detected with the Laplacian of Gaussian (LoG) algorithm. Parameters used for these preprocessing steps can be found in Table S1.

To calculate the intensity of a fluorescent focus, the focus area was determined by the resulting coordinates and radius of the LoG detection. The area of the focus was defined by a circle centered on the xy coordinate of the detection with radius = 2*r*, with *r* being the LoG determined spot radius. The pixel intensities within this circle were summed to define the focus intensity.

#### Carboxysome localization heatmaps

The localization coordinates of detected carboxysome foci and cell masks were used to make localization density heatmaps with the spideymaps tool (https://github.com/BiteenMatlab/spideymaps) in Python. For each segmented cell, morphological skeletonization was used to define the midline bisecting the cell lengthwise. Inspired by the *colicoords* package (55), the Cartesian coordinates of each focus (*x*, *y*) were re-parameterized into a three-coordinate system: *r*, *l*, and ϕ. *r* is the radial distance from the midline. *l* is the longitudinal distance along the length of the cell found from projection onto the midline. ϕ is the angle measured relative to the midline in the polar regions. *r* and *l* were divided by the average cell width and total length, respectively, to calculate *r*_rel_ and *l*_rel_ so that all localizations share a common frame of reference. Localizations were binned based on *r*_rel_, *l*_rel_, and ϕ. Because this approach produces unequal bin areas, bin areas were calculated for each bin in every cell. Localization counts and associated bin areas were summed across all cells. Areas and counts were symmetrized fourfold by adding counts and areas from symmetrically equivalent bins. The summed localization counts were divided by the summed areas to calculate average localization densities (counts per pixel [2]). Count densities were converted to probability densities by dividing by the total number of counts across all cells and multiplying by the number of cells. Probability density maps are reported with units of µm^−2^.

To generate representative cells for visualization, masks for individual cells were aligned and summed. Fifty percent of the max of summed masks was used as a threshold to create representative masks. The midlines and outlines of the representative masks were used to create the bin shapes for the figures.

#### McdA distribution

To analyze the localization of mNG-McdA in single cells, we implemented the same pixel intensity distribution analysis used previously (56). Briefly, the intensity of all the pixels in a cell was normalized by the minimum and maximum pixel intensity values in the corresponding cell: *I_n_* = (*I − I*_min_)/(*I*_max _*− I*_min_). These normalized pixel intensities were then binned to generate histograms that represent the localization pattern of the protein. To calculate the oscillation score, we determined the fraction of pixels with a normalized intensity below a threshold value, *I_n_* < 0.5 for each cell.

Drift-corrected time-lapse image stacks were used to generate kymographs along the cell length. In Fiji (57), a line was manually drawn along the long axis of a cell. The multi-kymograph tool was used to extract the intensity profile with an 11-pixel (660 nm) width. For visualization, the intensity profile at each time point of the kymograph was normalized to minimum and maximum intensity values within the corresponding time point. This post-processing removed the effects of photobleaching in the visualization of mNG-McdA.

#### Nucleoid morphology

To determine the nucleoid region within the cell, the cell mask was used to determine the pixel intensities within the inner rim of the cell boundary. The distribution of the inner rim pixel intensities was fit to a Gaussian distribution to determine the average intensity of the cytoplasmic region of the cell. This intensity was subtracted across all pixels in the cell. A Gaussian blur (standard deviation of Gaussian = 66 nm) was applied to the background-subtracted image. Next, the nucleoid region was detected using Yen’s thresholding (58). The pixels in the resulting region were considered the nucleoid. To calculate the nucleoid compaction score, *s*_comp_, we subtracted the ratio of the nucleoid area to the total cell area from 1: *s*_comp_ = 1 – (*A*_nuc_/*A*_cell_).

#### Pearson’s correlation analysis

To calculate single-cell Pearson correlation coefficients, first, cell masks were used to extract the pixel intensities for each cell across different color channels. The Pearson correlation coefficients (59) were then calculated for the pixel intensities across corresponding yellow (McdA) and cyan (carboxysomes) channels. McdB focus Pearson correlation coefficients were calculated using the JACoP plugin in FIJI on full fields of view from six different biological replicates using the corresponding McdB and carboxysome channels.

## SUPPLEMENTAL MATERIAL

Supplemental material mbio.01919-25-s0001.pdf Supplemental figures, tables, and video legends.

Video S1 mbio.01919-25-s0002.avi Carboxysomes in exponential phase cells.

Video S2 mbio.01919-25-s0003.avi Carboxysomes are less dynamic in stationary phase cells.

Video S3 mbio.01919-25-s0004.avi McdA robustly oscillates in exponential cells.

Video S4 mbio.01919-25-s0005.avi The McdA oscillation becomes diffuse in stationary phase cells.

Video S5 mbio.01919-25-s0006.avi McdA oscillates over the ciprofloxacin-compacted nucleoid.

ASM does not own the copyrights to Supplemental Material that may be linked to, or accessed through, an article. The authors have granted ASM a non-exclusive, world-wide license to publish the Supplemental Material files. Please contact the corresponding author directly for reuse.
