# Supplementary material for: Nucleoid compaction influences carboxysome localization and dynamics in Synechococcus elongatus PCC 7942
Source: mBio. 2025 Aug 21;16(10):e01919-25. doi: 10.1128/mbio.01919-25 (PMC12506020; doi:10.1128/mbio.01919-25)
Supplement: Supplemental material — Supplemental figures, tables, and video legends. [file mbio.01919-25-s0001.pdf]

SUPPLEMENTAL FIGURES

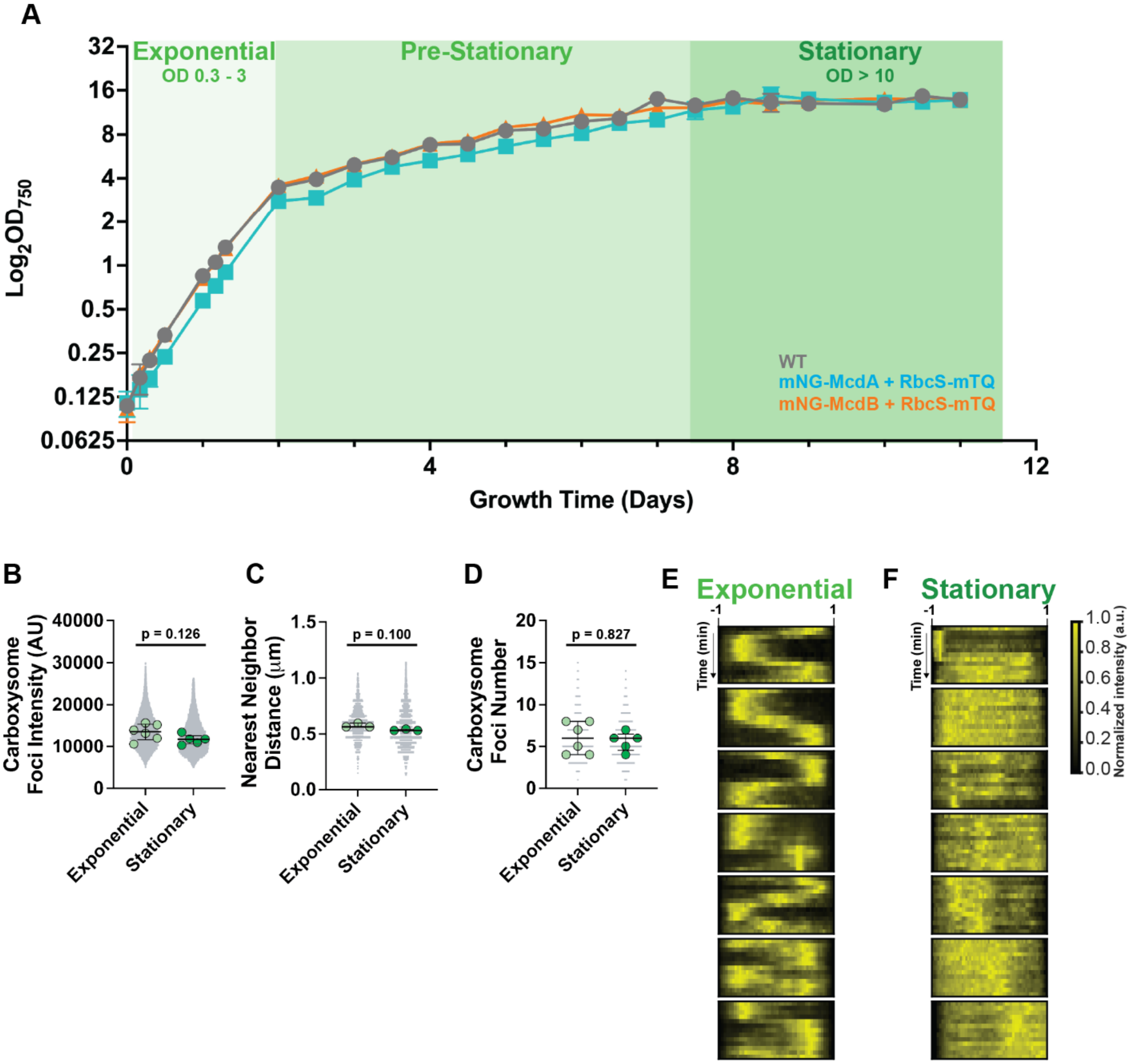

Figure S1 - (A) Growth curve of WT *S. elongatus* and the indicated fluorescently labeled strains. Fluorescent labeling does not significantly affect growth rate. n = 3 biological replicates per strain. Exponential phase is defined as OD_{750nm} 0.3 - 3. Stationary is defined as OD_{750nm} > 10. **(B)** Carboxysome foci intensity. Significance from Mann-Whitney test, (exponential) n = 6 biological replicates and (stationary) n = 5 biological replicates, >200 cells per replicate, bars show median and interquartile range. **(C)** Nearest neighbor distance between carboxysomes. Significance from Mann-Whitney test, (exponential) n = 6 biological replicates and (stationary) n = 5 biological replicates, >200 cells per replicate, bars show median and interquartile range. **(D)** Carboxysome number per cell. Significance from Mann-Whitney test, (exponential) n = 6 biological replicates and (stationary) n = 5 biological replicates, >200 cells cells per replicate, bars show median and interquartile range. **(E)** Representative kymographs of McdA dynamics in exponential phase cells. **(F)** Representative kymographs of mNG-McdA dynamics in stationary phase cells. Long-axis is cell length, short-axis is time, 0 to 60 minutes.

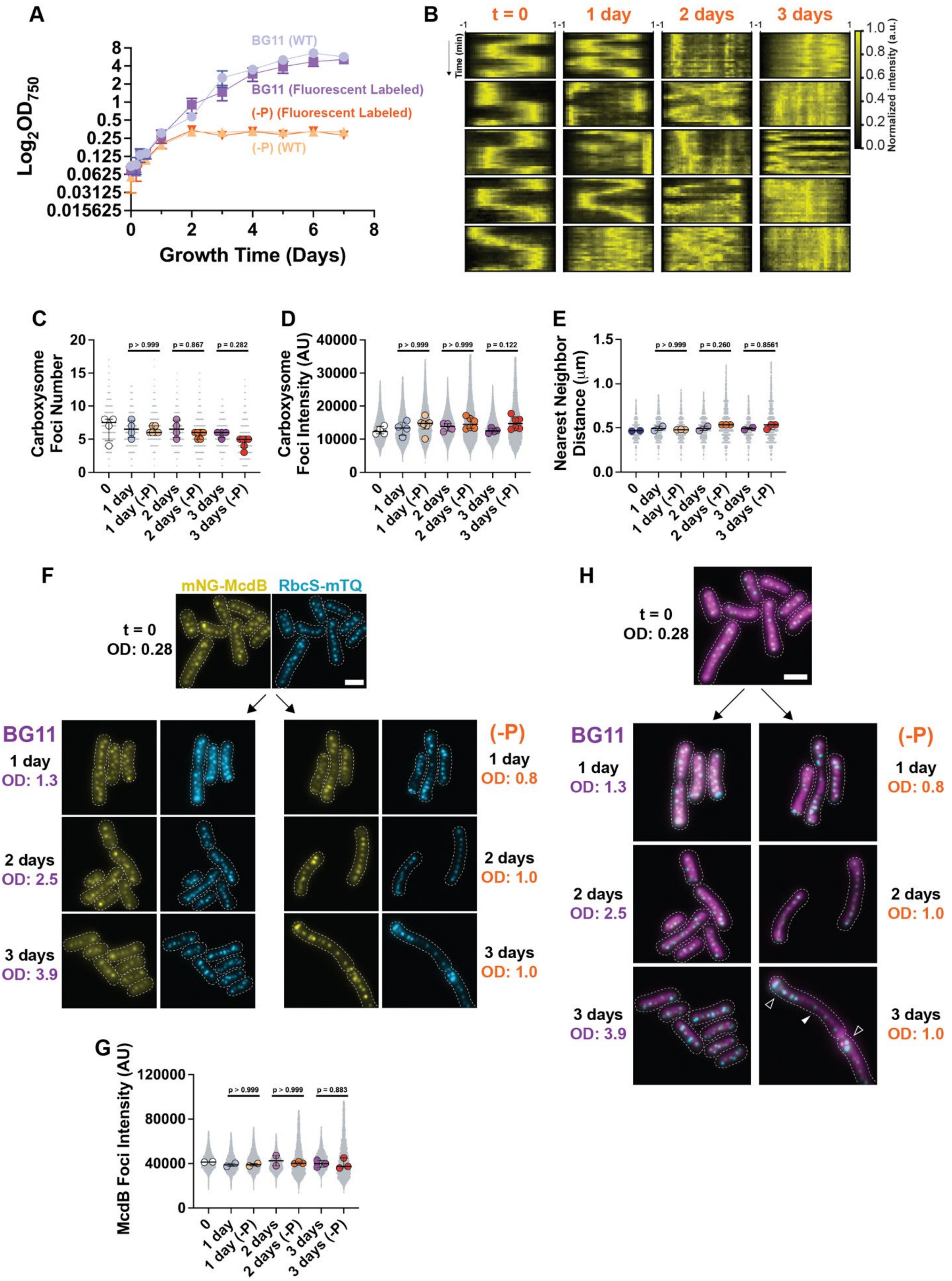

A
Log$_2$OD$_{750}$
8
4
2
1
0.5
0.25
0.125
0.0625
0.03125
0.015625
0
2
4
6
8
Growth Time (Days)
BG11 (WT)
BG11 (Fluorescent Labeled)
(-P) (Fluorescent Labeled)
(-P) (WT)
B
t = 0
1 day
2 days
3 days
-1
1-1
1-1
1-1
1
Time (min)
Normalized intensity (a.u.)
1.0
0.8
0.6
0.4
0.2
0.0
C
Carboxysome Foci Number
20
15
10
5
0
p > 0.999
p = 0.867
p = 0.282
0
1 day
1 day (-P)
2 days
2 days (-P)
3 days
3 days (-P)
D
Carboxysome Foci Intensity (AU)
40000
30000
20000
10000
0
p > 0.999
p > 0.999
p = 0.122
0
1 day
1 day (-P)
2 days
2 days (-P)
3 days
3 days (-P)
E
Nearest Neighbor Distance (μm)
1.5
1.0
0.5
0.0
p > 0.999
p = 0.260
p = 0.8561
0
1 day
1 day (-P)
2 days
2 days (-P)
3 days
3 days (-P)
F
mNG-McdB
RbcS-mTQ
t = 0
OD: 0.28
BG11
(-P)
1 day
OD: 1.3
1 day
OD: 0.8
2 days
OD: 2.5
2 days
OD: 1.0
3 days
OD: 3.9
3 days
OD: 1.0
G
McdB Foci Intensity (AU)
120000
80000
40000
0
p > 0.999
p > 0.999
p = 0.883
0
1 day
1 day (-P)
2 days
2 days (-P)
3 days
3 days (-P)
H
t = 0
OD: 0.28
BG11
(-P)
1 day
OD: 1.3
1 day
OD: 0.8
2 days
OD: 2.5
2 days
OD: 1.0
3 days
OD: 3.9
3 days
OD: 1.0

Figure S2 - (A) Growth curve of WT *S. elongatus* and fluorescently labeled strains grown in BG11 and BG11 lacking phosphate (-P). Fluorescent labeling does not significantly affect growth rate. n = 3 biological replicates per treatment. **(B)** Representative kymographs of mNG-McdA dynamics in phosphate deprivation conditions over 3 days. Long-axis is cell length, short-axis is time, 0 to 60 minutes. **(C)** Carboxysome number per cell. Significance from Kruskall-Wallis and Dunn's multiple comparisons, (BG11) n = 4 biological replicates and (-P) n = 6 replicates, >200 cells per replicate, bars show median and interquartile range. **(D)** Carboxysome foci intensity. Significance from Kruskall-Wallis and Dunn's multiple comparisons, (BG11) n = 4 replicates and (-P) n = 6 replicates, >200 cells per replicate, bars show median and interquartile range. **(E)** Distance between carboxysome and its nearest carboxysome neighbor. Significance from Kruskall-Wallis and Dunn's multiple comparisons, (BG11) n = 4 replicates and (-P) n = 6 replicates, >200 cells per replicate, bars show median and interquartile range. **(F)** Microscopy images of mNG-McdB in cells grown in BG11 and phosphate-limited BG11 over a 3-day time course. Carboxysomes are in cyan. Dotted white line shows cells' boundaries from phase contrast. **(G)** McdB foci intensity. Significance from Kruskall-Wallis and Dunn's multiple comparisons, (BG11) n = 2 biological replicates and (-P) n = 3 biological replicates, >200 cells per replicate, bars show median and interquartile range. **(H)** Merged microscopy images of DAPI stained nucleoid and carboxysomes (cyan) in cells grown in BG11 and phosphate-limited BG11 over a 3-day time course. White arrows indicate areas where carboxysomes are singly spaced on a compacted nucleoid and clumped in expanded regions. (scale bar 2 μm).

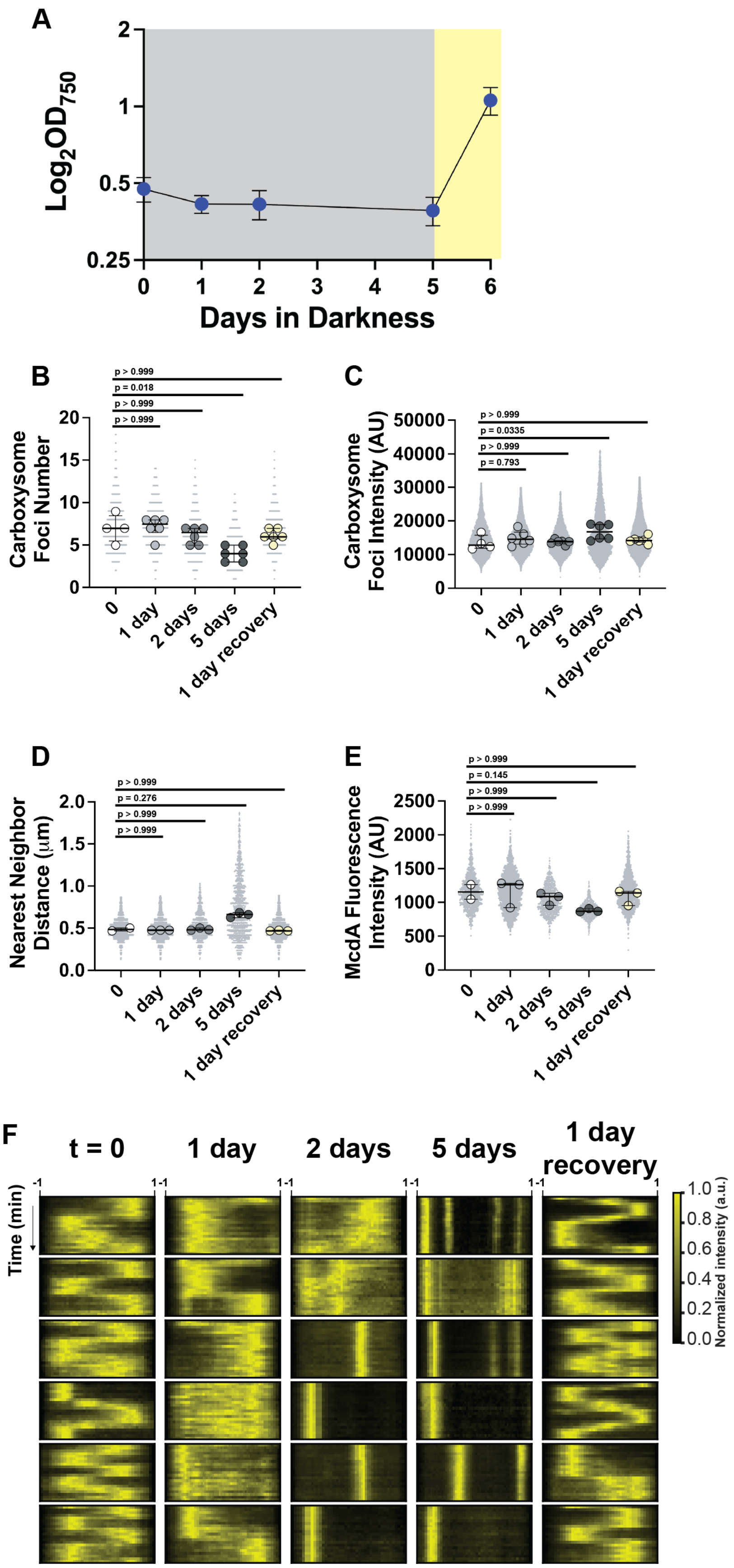

A
Log$_2$OD$_{750}$
2
1
0.5
0.25
0 1 2 3 4 5 6
Days in Darkness
B
Carboxysome Foci Number
p > 0.999
p = 0.018
p > 0.999
p > 0.999
0 1 day 2 days 5 days 1 day recovery
C
Carboxysome Foci Intensity (AU)
p > 0.999
p = 0.0335
p > 0.999
p = 0.793
D
Nearest Neighbor Distance (μm)
p > 0.999
p = 0.276
p > 0.999
p > 0.999
E
McdA Fluorescence Intensity (AU)
p > 0.999
p = 0.145
p > 0.999
p > 0.999
F
t = 0
1 day
2 days
5 days
1 day recovery
Time (min)
Normalized intensity (a.u.)

Figure S3 - (A) Growth curve of *S. elongatus* incubated in continuous darkness for 5 days then recovered in continuous light for 1 day. n = 4 biological replicates. **(B)** Carboxysome number per cell. Significance from Kruskall-Wallis and Dunn's multiple comparisons, 1 t = 0, n = 4 biological replicates; day 1 – 5 and 1 day recovery, n = 6 biological replicates per time point, >200 cells per replicate, bars show median and interquartile range. **(C)** Carboxysome foci intensity. Significance from Kruskall-Wallis and Dunn's multiple comparisons, 1 t = 0, n = 4 biological replicates; day 1 – 5 and 1 day recovery, n = 6 biological replicates per time point, >200 cells per replicate, bars show median and interquartile range. **(D)** Nearest neighbor distance between carboxysomes. Significance from Kruskall-Wallis and Dunn's multiple comparisons, t = 0, n = 4 biological replicates; day 1 – 5 and 1 day recovery, n = 6 biological replicates per time point, >200 cells per replicate, bars show median and interquartile range. **(E)** mNG-McdA whole cell fluorescence intensity normalized by cell length. Significance from Kruskall-Wallis and Dunn's multiple comparisons, 1 t = 0, n = 2 biological replicates; day 1 – 5 and 1 day recovery, n = 3 biological replicates per time point, >200 cells per replicate, bars show median and interquartile range. **(F)** Representative kymographs of McdA dynamics in prolonged darkness conditions over 5 days and 1 day of growth recovery in light. Long-axis is cell length, short-axis is time, 0 to 60 minutes. (scale bar 2 μm).

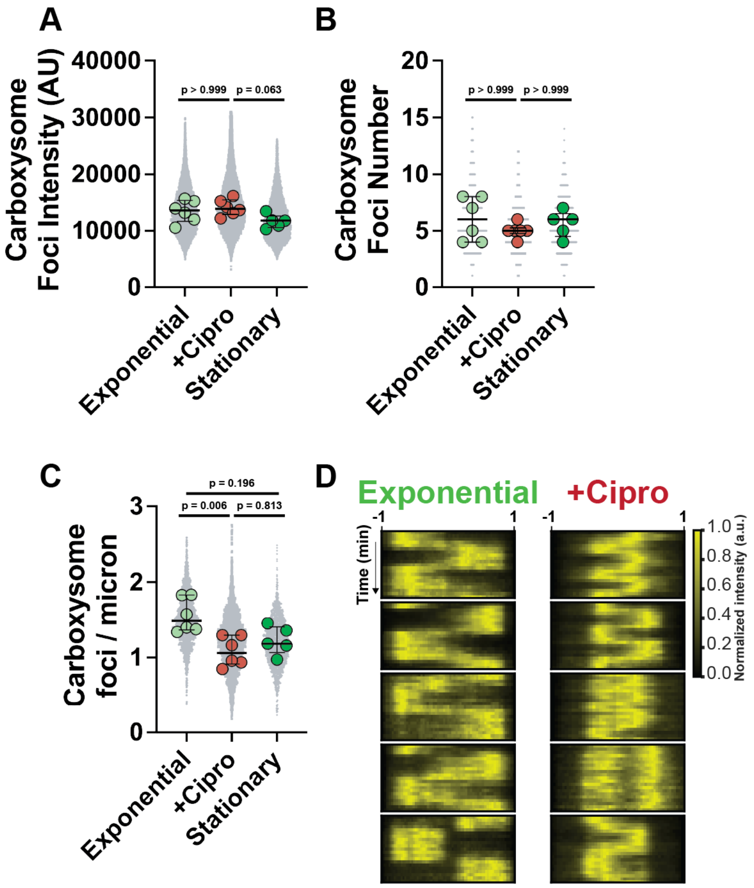

Figure S4 - (A) Carboxysome foci intensity. Significance from Kruskall-Wallis and Dunn's multiple comparisons, (exponential) n = 6 biological replicates, (cipro) n = 6 biological replicates, and (stationary) n = 5 biological replicates, >200 cells per replicate, bars show median and interquartile range. **(B)** Carboxysome number per cell. Significance from Kruskall-Wallis and Dunn's multiple comparisons, (exponential) n = 6 biological replicates, (cipro) n = 6 biological replicates, and (stationary) n = 5 biological replicates, >200 cells per replicate, bars show median and interquartile range. **(C)** Carboxysomes per unit of cell length, calculated by dividing the number of foci by cell length. Significance from Kruskall-Wallis and Dunn's multiple comparisons, (exponential) n = 6 biological replicates, (cipro) n = 6 biological replicates, and (stationary) n = 5 biological replicates, >200 cells per replicate, bars show median and interquartile range. **(D)** Representative kymographs of McdA dyanmics in exponential phase cells with and without ciprofloxacin treatment. Long-axis is cell length, short-axis is time, 0 to 60 minutes.

SUPPLEMENTARY TABLE 1

Fluorescent Focus Detection Parameters

Carboxysomes:

Background Subtraction	restoration.rolling_ball(cell_fluor,radius = 3)
Image Denoise	filters.unsharp_mask(cell_fluor_bg, radius = 2, amount = 5)
Add Gaussian Blur	filter.gaussian(cell_fluor_sharp, 0.01)
Carboxysome Detection	min_sigma = 2.75, max_sigma = 3, threshold = 0.005, overlap = 0.75

McdA Foci:

Background Subtraction	restoration.rolling_ball(cell_fluor,radius = 5)
Image Denoise	filters.unsharp_mask(cell_fluor_bg, radius = 3, amount = 2)
Add Gaussian Blur	filter.gaussian(cell_fluor_sharp, 0.5)
McdA Foci Detection	min_sigma = 1.5, max_sigma = 5, threshold = 0.01, overlap = 0.95

McdB Foci:

Background Subtraction	restoration.rolling_ball(cell_fluor,radius = 2.5)
Image Denoise	filters.unsharp_mask(cell_fluor_bg, radius = 1.5, amount = 1)
Add Gaussian Blur	filter.gaussian(cell_fluor_sharp, 0.01)
McdB Foci Detection	min_sigma = 1.5, max_sigma = 5, threshold = 0.003, overlap = 0.75
